# Structure of an *E. coli* integral membrane sulfurtransferase and its structural transition upon SCN^−^ binding defined by EPR-based hybrid method

**DOI:** 10.1038/srep20025

**Published:** 2016-01-28

**Authors:** Shenglong Ling, Wei Wang, Lu Yu, Junhui Peng, Xiaoying Cai, Ying Xiong, Zahra Hayati, Longhua Zhang, Zhiyong Zhang, Likai Song, Changlin Tian

**Affiliations:** 1 National Laboratory for Physical Science at Microscale, School of Life Science, University of Science and Technology of China, and High Magnetic Field Laboratory, Chinese Academy of Sciences, Hefei, 230027, P. R. China; 2 National High Magnetic Field Laboratory, Florida State University, Tallahassee, FL, 32310, U.S.A

## Abstract

Electron paramagnetic resonance (EPR)-based hybrid experimental and computational approaches were applied to determine the structure of a full-length *E. coli* integral membrane sulfurtransferase, dimeric YgaP, and its structural and dynamic changes upon ligand binding. The solution NMR structures of the YgaP transmembrane domain (TMD) and cytosolic catalytic rhodanese domain were reported recently, but the tertiary fold of full-length YgaP was not yet available. Here, systematic site-specific EPR analysis defined a helix-loop-helix secondary structure of the YagP-TMD monomers using mobility, accessibility and membrane immersion measurements. The tertiary folds of dimeric YgaP-TMD and full-length YgaP in detergent micelles were determined through inter- and intra-monomer distance mapping and rigid-body computation. Further EPR analysis demonstrated the tight packing of the two YgaP second transmembrane helices upon binding of the catalytic product SCN^−^, which provides insight into the thiocyanate exportation mechanism of YgaP in the *E. coli* membrane.

Membrane proteins play essential roles in material transportation, signal transduction, and energy metabolism across the cell membrane. Structural studies of membrane proteins provide a detailed understanding of their functional mechanisms. X-ray diffraction and cryo-electron microscopy determine membrane protein structures at high resolution, whereas magnetic resonance methods can provide both structure and dynamic information for membrane proteins^1^,^2^. However, nuclear magnetic resonance (NMR) is only capable of characterizing small size membrane proteins, which has limited its application considerably. Electron paramagnetic resonance (EPR) has almost three order of magnitude more sensitivity than NMR, and the combined method of site-directed spin labeling (SDSL) and EPR enables structural constraint collections for membrane proteins with unlimited size during their structural characterization using EPR^3^. For the past two decades, combinational SDSL and EPR have been widely applied in dynamic, secondary structure and distance analyses of protein-protein interactions as well as in protein structure-function correlation studies^3^,^4^,^5^,^6^,^7^,^8^. Moreover, the capability of EPR for use in *de novo* high-resolution soluble protein structure determination has been demonstrated recently, using EPR-derived constraints along with a computational approach^9^,^10^,^11^. Here, we report the use of this method to determine the structure of a full-length membrane protein – YgaP.

Dimeric YgaP is an *E. coli* integral membrane sulfurtransferase^12^,^13^. Each monomer contains a cytosolic catalytic rhodanese domain (1–107) and a transmembrane domain (TMD; 117–174), which was predicted to have two helices (Supplementary Figure 1a). The cytosolic rhodanese domain was reported to catalyze the transfer of a sulfur atom (S) from thiosulfate (−S=SO3^2−^) to the toxic cyanide (CN) group, resulting in non-toxic thiocyanate (SCN) and sulfite (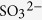)^14^,^15^. Biochemical and structural studies of rhodanese domains from bacteria and eukaryotic cells have elucidated their tertiary structures and the locations of the conserved cysteine residues involved in sulfur transfer catalysis^15^,^16^,^17^,^18^. Eight genes encoding proteins with rhodanese domains were reported in the *E. coli* genome^19^, but YgaP is the only one containing another transmembrane domain, which was hypothesized to export the non-toxic thiocyanate (SCN^−^) across the membrane, marking the end of the cyanate detoxification process in *E. coli*^12^.

Recently, the solution NMR tertiary structure of the cytosolic YgaP rhodanese domain was reported independently by our group^20^ and Eichmann *et al.*^21^. In the report of Eichmann group, the tertiary fold of full-length YgaP is not available, due to the lack of long distance constraints between the rhodanese domain and the transmembrane domain, although the structures of these two domains were both determined using solution NMR. Chemical shift perturbations of full-length YgaP with different concentrations of sodium thiosulfate (NaSSO_4_) were observed for the ^15^N-^1^H moieties of residues located at the second transmembrane helix, whereas no obvious chemical shift perturbations were observed in the YgaP rhodanese domain upon the addition of SCN^−^. These observations indicated that the YgaP-TMD may participate in the exportation of SCN^−^ after the cyanate detoxification process^21^.

In the present report, an EPR-based hybrid experimental and computational approach was applied to determine the three-dimensional topological structure of full-length dimeric YgaP in DPC micelles. Systematic scanning continuous wave (CW)-EPR and power saturation experiments were implemented for site-specific mobility and accessibility analyses, resulting in the characterization of the secondary structures of the YgaP-TMD. Inter- and intra-monomer distance constraints were also acquired using combinational CW-EPR and the double electron-electron resonance (DEER) approach. For the same sample, distances between spin pairs in the range of around 8–25 Å can be measured using CW-EPR, while distances between spin pairs in the range of 20–80 Å can be measured using DEER EPR method. Subsequently, in conjunction with the solution NMR structure of the rhodanese domain determined in our laboratory and rigid-body computational methods, the dimeric full-length YgaP structural fold was achieved in DPC micelles using the EPR distance constraints. Moreover, the CW-EPR mobility, accessibility and distance analyses of YgaP in the absence or presence of SCN^−^ lead to a hypothesized transmembrane helical packing model of the dimeric full-length YgaP-TMD upon SCN^−^ binding.

## Results

### Secondary structure of the transmembrane domain of YgaP defined by EPR mobility and accessibility analyses

**Figure 1:**
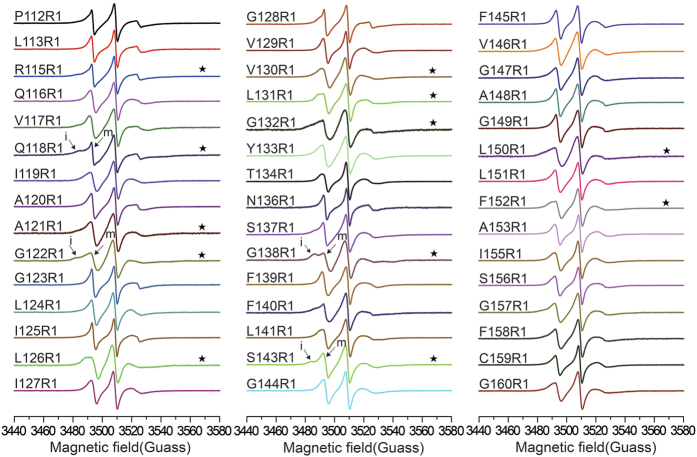
CW-EPR spectra of 45 spin-labeled YgaP variants in TMD (from Pro112 to Gly160, except M114, V135, L142, and G154) at 298 K. Asterisks indicate spectra that exhibit multiple components. Each spectrum was normalized by the height of the central peak. “m” and “i” represent the “mobilized” and “immobilized” components, respectively.

The dimeric full-length YgaP in detergent micelles for EPR studies were verified using SDS-PAGE analysis in the presence of cross-linking reagents (Supplementary Figure 1b). The topology prediction of full-length YgaP using TMHMM demonstrated that the TMD (from I119 to D166) contained two putative transmembrane helices, as shown in Supplementarry Figure 1a. A total of 45 cysteine variants (P112-G160, except M114, V135, L142, and G154, which resulted in poor expression after cysteine mutations) covering the putative YgaP-TMD sequence were spin labeled using methanethiosulfonate (MTSL)^22^, followed by CW-EPR spectra collection at 298 K. The circular dichroism (CD) spectra of cysless variant (YgaP C64S&C159S) and spin-labeled variants (represented by YgaP A120R1&S143R1) are highly similar to the wild type protein in the 198, 208 and 223 nm region (Supplementary Figure 2), indicating that there are no significant structure perturbations caused by mutagenesis.

**Figure 2:**
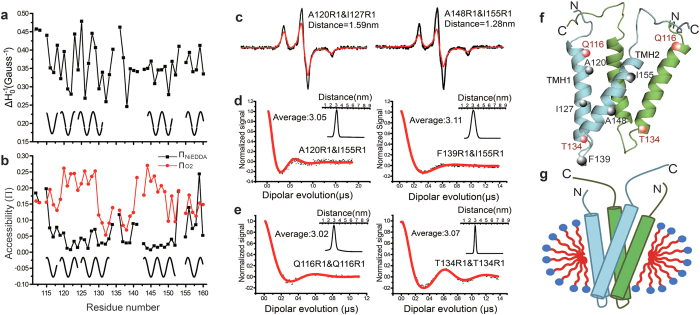
Structural characterization of dimeric YgaP-TMD using EPR-derived constraints. (**a**) A plot of the reciprocal of the central resonance line width, 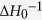, of the CW-EPR spectrum versus residue number. (**b**) Accessibility parameters of NiEDDA (black squares) and O_2_ (red circles) plotted versus the residue number. The periodicity of the fluctuation in the 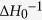 values indicates the α-helix secondary structure. (**c**) Intra-monomeric CW-EPR distance measurements of double labeled YgaP samplesat 150 K. The black line represents the sum of the spectra of two single-labeled samples, while the red line represents the spectrum of the double-labeled samples. (**d,e**) Intra-and Inter-monomeric DEER-EPR distance measurements of the double-labeled YgaP samples at 65 K. The red line was the best fit for the normalized signal versus the dipolar evolution time. (**f**) Structure of dimeric YgaP-TMD calculated using EPR derived information. The highlighted residues were MTSL labeled for distance measurements. (**g**) Corresponding topological model of dimeric YgaP-TMD in DPC micelles. Each monomer of YgaP is colored cyan or green.

As shown in Fig. 1, all CW-EPR spectra of spin-labeled YgaP in DPC micelles exhibited three-line spectra typical for nitroxide based spin probes. Although the majority of the residues exhibited only one motional component, certain residues had multiple motional states (Fig. 1, marked with an asterisk), indicating that the side chains of these residues may have different motional or conformational states (a mobilized (m) or immobilized (i) component). The inverse of the central m_I=0_ resonance line width (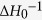) is determined primarily by the degree of averaging of the anisotropic g tensor. 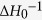 is normally used as a comprehensive descriptor of nitroxide mobility and the corresponding structure features, with larger 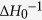 values representing the higher mobility of a specific residue^5^. Here, large mobility variations were observed for residues in the YgaP-TMD. Among the residues with low 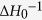 values shown in Fig. 2a, R115, Q118, A121, G122, L126, V130, G132, G138, L150 and F152 had multiple dynamic components (Fig. 1). Overall, the relatively high degree of fluctuation in the 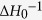 values was observed for residues in the YgaP-TMD, especially for the first predicted transmembrane segment (R114-V135). For residues on the second predicted transmembrane segment (S142-G160), the EPR mobility of the spin-labeled residues showed smaller fluctuations along the sequence, indicating that this domain is located at the interface between monomers. Moreover, a periodicity of the fluctuations in the 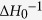 was observed for multiple regions (R114-V135 and S142-G160), indicating two helical structures of the YgaP-TMD^23^. Since YgaP formed homo-dimers in detergent micelles (Supplementary Figure 1b), the residue sites with lower mobility or 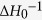 values (e.g. V117, Q118, G122, L126, L131, G132, S137, G138, L141, S143, L150, and F152) may play roles in the inter-helical interactions of the dimeric YgaP-TMDs.

Moreover, to assist membrane protein topology analysis, accessibility parameters of the spin-labeled residues were obtained from the collision frequency of the nitroxide radical probe with paramagnetic relaxation reagents^24^. Here, both hydrophobic paramagnetic relaxation reagent oxygen (O_2_ in air) and hydrophilic reagent NiEDDA were used to facilitate accessibility evaluation^25^. As shown in supplementary Figure 3, the accessibility parameter (Π, proportional to the collision frequency of nitroxide with either O_2_ or NiEDDA) can be estimated using power saturation EPR techniques^24^. Systematic scanning Π_O2_ and Π_NiEDDA_ of residues in the YgaP-TMD (Fig. 2b) were collected to provide topology information^26^,^27^. As illustrated for putative YgaP-TMH1 (Fig. 2b), the Π_NiEDDA_ values progressively decreased (from R115 to V117), followed by a flat region with low Π_NiEDDA_ values (from Q118 to G132), then the Π_NiEDDA_ values increased from residues Y133 to N136. This decrease-flat-increase pattern of Π_NiEDDA_ values indicated a highly hydrophobic core region (Q118-G132) in this region and the two flanking regions located in the hydrophilic/hydrophobic interface part of the detergent micelles. A similar pattern was observed for the putative YgaP-TMH2, which had low Π_NiEDDA_ values from S143 to F152, indicating a hydrophobic environment. The Π_O2_ values in Fig. 2b showed a pattern complementary to the Π_NiEDDA_ values in YgaP-TMH1 and TMH2. Therefore, two hydrophobic regions (R115 to V130 for TMH1 andL141 to F152 for TMH2) could be derived, which was also validated by the immersion depth parameter, Φ (Supplementary Figure 4). For residues other than these two regions, which were observed to have low Π_O2_ and high Π_NiEDDA_ values, a loop region between two TMHs could be assigned connecting two hydrophilic YgaP-TMHs. The α-helical periodicity for the two transmembrane helices could also be verified by both the Π_O2_ and Π_NiEDDA_ accessibility data. Taken together, the periodicity of both 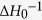 and accessibility parameters (Π_O2_ and Π_NiEDDA_) along the primary sequence of YgaP-TMD demonstrated that this domain contained two transmembrane helices: TMH1 (M114-V135) and TMH2 (L142-G160), providing the basis for further tertiary structure characterization of the dimeric YgaP-TMD.

### Tertiary structural fold of full-length dimeric YgaP-TMD determined through EPR distance measurements

To obtain the tertiary structure of the YgaP-TMD, distance constraints between the two derived YgaP-TMHs in the same monomer or between two monomers were required. In total, 30 pairs of double cysteine substitution mutants of full-length dimeric YgaP were generated for distance measurements in DPC micelles. Among them, 12 pairs were prepared for distance measurements using CW-EPR (Fig. 2c) or DEER (Fig. 2d) to obtain intra-monomer constraints between the two YgaP-TMHs. As shown in Fig. 2c and Supplementary Figure 5, spectrum broadening was observed for doubly labeled proteins as well as singly labeled proteins, indicating dipolar coupling between the two spin labels separated within the range of 8–25 Å^28^,^29^. The Fourier deconvolution method was applied to obtain the inter-spin distance and its distribution^30^. The EPR derived distance distributions can reflect the protein fluctuation dynamics and the flexibility of spin label side chain^31^. As shown in Fig. 2c, the distance between residues A120 and I127 was measured as 1.59 nm, and the distance between residues A148 and I155 was measured as 1.28 nm. For intra-monomer distances longer than 25 Å, DEER measurements were conducted to derive the distance values and distributions^30^,^32^,^33^. As shown in Fig. 2d, the intra-monomer distances were found to be 3.05 nm between spin pairs A120-I155 and 3.11 nm for F139-I155. Eight CW-EPR-derived distances and four DEER-derived distances were obtained (Supplementary Table 1). All the measured distances had relative narrow distance distributions, indicating a rather ordered and rigid conformation for the spin-labeled residues on the transmembrane domain.

The obtained distance constraints were applied for structure calculation of the transmembrane domain of full-length dimeric YgaP. Briefly, the measured distances between spin labels were translated into distances between β−carbons (C_β_) of residues using a “motion-on-a-cone” model of the R1 site relative to the β−carbons (C_β_)^11^,^34^. The “motion-on-a-cone” model yields a predicted distribution for the difference between the distance separating the spin labels (d_SL_) and that separating the two corresponding β carbons (d_Cβ_), providing a constraint in the form of a predicted range for d_Cβ_. Previous studies have demonstrated the fidelity of this method for protein structure characterization using EPR distance data^11^.

Based on the measured intra-monomer distance constraints, the topological structure of the monomeric YgaP-TMD was first assessed. The preliminary model of YgaP-TMD containing two EPR derived rigid α-helices (TMH1: R114-V135 and TMH2: L142-G160) linked by a flexible loop was used as the starting point. Then, the intra-monomer distances constraints were applied for structure calculation using XPLOR-NIH^35^. The detailed procedure can be found in the Methods. In brief, the two α-helices were treated as rigid bodies. The conjoined rigid body/torsion angle/Cartesian dynamics simulated annealing protocol was used, in which the rigid bodies were allowed to translate and rotate freely while connected by a flexible linker with Cartesian degrees of freedom^36^,^37^. As an intermediate protein model, the top 20 structural model of monomeric YgaP-TMD from a total of 96 models were illustrated in Supplementary Table 2. Structural alignment of our monomeric YgaP-TMD with the NMR structure of YgaP-TMD solved by Eichmann *et al.* (PDB code 2MPN) showed fairly similar topologies despite several structural differences (Supplementary Figure 6). The calculated monomeric YgaP-TMD structure was less compact compared with the NMR structure (PDB code 2MPN), as reflected by a deviated transmembrane helix in the N-terminus.

In the next computational step, the monomeric YgaP-TMD was treated as a rigid body, and a total of 9 inter-monomer distance constraints (Fig. 2c and Supplementary Figure 7) were applied to translate and rotate one monomer with respect to the other. The top 100 structures were derived from 1,000 models, and the average pair-wise rmsd deviation of the backbone Cα reached 1.81 Å (Supplementary Table 3). As shown in Fig. 2f, the YgaP-TMD was composed of four intertwined transmembrane helices. The TMH2 regions from each monomer interfaced with one another. Several residues on TMH2 (e.g., F145, G149, A153 and G157) were located at the helix-helix packing interface, whereas residues A120, L124, G128 and L131 on TMH1 were interior-facing, which was consistent with their mobility (Fig. 2a) and accessibility (Fig. 2b) analyses. Each monomer of the YgaP-TMD contained two slightly tilted (i.e., ~20°) α-helices. The C-terminus of TMH1 (residues L131, G132, Y133, and T134) and the N-terminus of TMH2 (residues L142, S143, and G144) were in close proximity. In contrast, the N-terminus of TMH1 (residues M114, R115, and Q116) and the C-terminus of TMH2 (residues F158, C159 and G160) were rather far apart.

### Conformation characterization of the full-length dimeric YgaP

**Figure 3:**
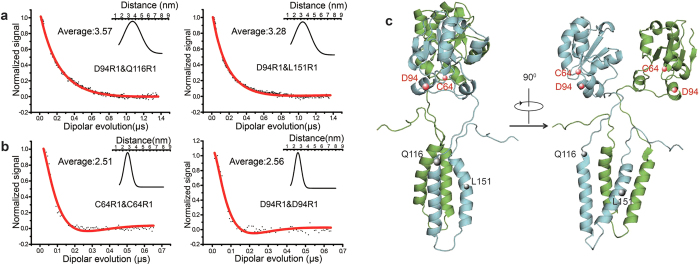
Structural characterization of full-length dimeric YgaP using EPR-derived constraints. (**a**) Intra-monomer (between TMD and the rhodanese domain) DEER distance measurements of dimeric full-length YgaP samples at 65 K. (**b**) Inter-monomer (between two TMDs) DEER distance measurements of dimeric full-length YgaP samples at 65 K. The red line represents the best fit of the normalized signal versus the dipolar evolution time. (**c**) The structural model of full-length YgaP, calculated from sparse long-distance constraints using XPLOR-NIH. Each monomer of YgaP is colored cyan and green.

The full-length YgaP protein from *E. coli* contains a cytosolic rhodanese domain and a transmembrane domain. Recently, the solution NMR structure of the YgaP rhodanese domain was revealed to adopt a typical rhodanese domain containing a α−β−α sandwich fold^20^. The detailed protocol used for structural model refinement of dimeric full-length YgaP was presented in Supplementary Figure 8. To characterize the conformation of dimeric full-length YgaP, a total of 5 intra-monomer (between TMD and the rhodanese domain, Fig. 3a and Supplementary Figure 9) and 4 inter-monomer (between two rhodanese domains, Fig. 3b) long-distance constraints were obtained using the DEER (Supplementary Table 1). Using the dimeric model of the YgaP-TMD (Fig. 2f) together with the solution NMR tertiary structure of the YgaP rhodanese domain, dimeric full-length YgaP was calculated using the XPLOR-NIH software package. A total of 16 models were well converged, and the final overall structural model of the YgaP homo-dimer is illustrated in Fig. 3c and Supplementary Figure 10 with a backbone Cα rmsd of 1.59 Å (Table 1). From the final model, the YgaP rhodanese domain was connected with the TMD by a long flexible linker (G100-V117), which likely contributed to the broad distribution of the intra-monomer DEER distance between the rhodanese domain and TMD (Fig. 3a).

### SCN^−^ binding decreases the mobility of TMH2 in full-length dimeric YgaP

YgaP was found to have sulfurtransferase activity and to play a crucial role in the detoxification of cyanide (CN^−^) by catalyzing the transfer of a sulfane sulfur from thiosulfate (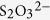) to cyanide, leading to the formation of the less toxic product, thiocyanate (SCN^−^)^21^. The sulfur transfer process has been characterized by various studies; however, the mechanism of the outward transportation of SCN^−^ remains unclear. Because YgaP is the only member of the rhodanese family with a transmembrane domain in *E. coli*^12^, it was suggested that YgaP may also play an essential role in the exportation of enzymatic product, SCN^−^ to the periplasm. Previous NMR titration experiments and DEER distance measurements suggested that SCN^−^ might induce conformational changes in the YgaP transmembrane domain, especially in TMH2^21^. However, YgaP mobility details and conformational changes upon SCN^−^ binding were still absent.

**Figure 4:**
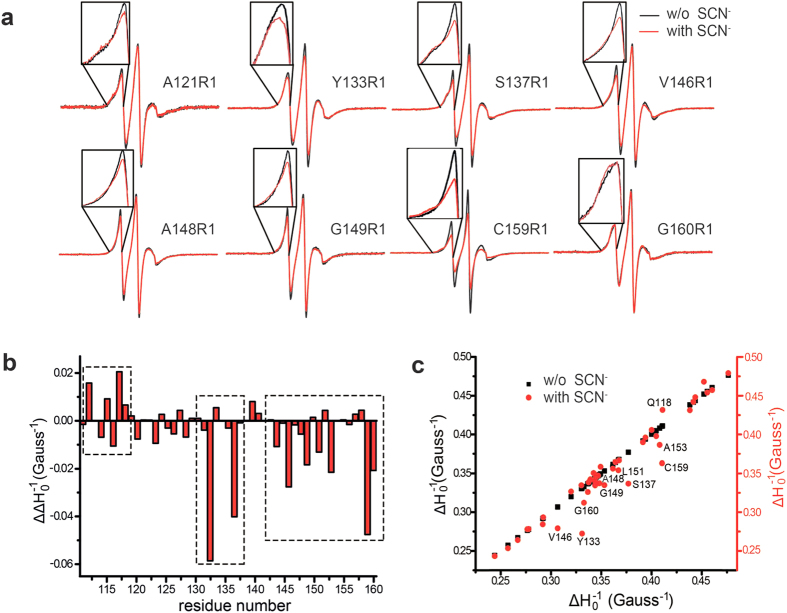
Mobility change of full-length YgaP upon SCN^−^ binding. (**a**) CW-EPR spectral overlap of YgaP variants (A121R1, Y133R1, S137R1, V146R1, A148R1, G149R1, C159R1, and G160R1) in the presence (red line) or absence of SCN^−^ (black line) at 298 K. Each spectrum was normalized by the intensity. (**b**) Histogram deviation analysis of 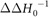 between the YgaP samples in the absence or presence of SCN^−^ plotted versus the residue number. (**c**) Correlation analysis of the central resonance line width reciprocal (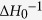) of the CW-EPR spectrum of YgaP in the absence (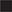) or presence of SCN^−^ (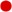). The residue sites with clear 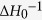 variations (labeled as red dots) were observed away from the line with a slope of 1.

To achieve YgaP mobility analysis or potential changes of EPR-derived mobility and accessibility, CW-EPR spectra and power saturation experiments were performed on a total of 45 sequential residues (P112-G160, except M114, V135, L142, and G154) in the YgaP-TMD in the presence or absence of SCN^−^, the catalytic product, at room temperature (Supplementary Figure 11). As shown in Fig. 4a, apparent spectrum broadening of variants A121R1, Y133R1, S137R1, V146R1, A148R1, G149R1, C159R1 and G160R1 was observed when SCN^−^ was added to the sample. Spectral broadening, especially when observed in low field resonances^5^, indicated a state of lower mobility for the R1 side chain on the transmembrane domain of full-length YgaP, which was apparently caused by SCN^−^ binding. Simultaneously, multiple motional states were observed in several CW-EPR spectra of the YgaP variants, both in the presence or absence of SCN^−^, including A121R1, S137R1, C159R1 and G160R1. However, the fractions of immobilized components in the CW-EPR spectra of these sites increased upon SCN^−^ addition (Fig. 4a), indicating that YgaP in the presence of SCN^−^ may prefer a more immobilized state in detergent micelles.

Furthermore, the mobility parameter 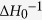 was extracted from the CW-EPR spectra to derive the mobility information of the YgaP-TMD upon the addition of SCN^−^. Changes in 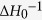 caused by SCN^−^ binding were summarized in the histogram of Fig. 4b. Despite minor deviations of 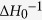 in most residues, pronounced 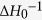 changes were observed in a small set of residue sites, including L113R1, Q118R1, Y133R1, S137R1, V146R1, G149R1, C159R1 and G160R1. These residues resided in three different regions: the N-terminus of TMH1, TMH2 and the loop regions connecting the two TMHs, as shown in the three dashed boxes in Fig. 4b. Furthermore, dramatically decreased 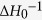 values were observed for residue sites in the loop and the TMH2 regions of YgaP, indicating markedly decreased mobility in the two regions. Moreover, correlation analyses of the derived 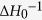 values in the absence or presence of SCN^−^ are plotted in Fig. 4c. In this plot, the residue sites with large deviations upon SCN^−^ addition reside largely on the second transmembrane helix (TMH2), particularly the second half of TMH2 (residues V146-G160). These observations indicated that the binding of SCN^−^ to the YgaP-TMD might lead to packing interactions between the second half of TMH2 and consequent decreased mobility.

### Conformational rearrangement of YgaP-TMD upon SCN^−^ binding

**Figure 5:**
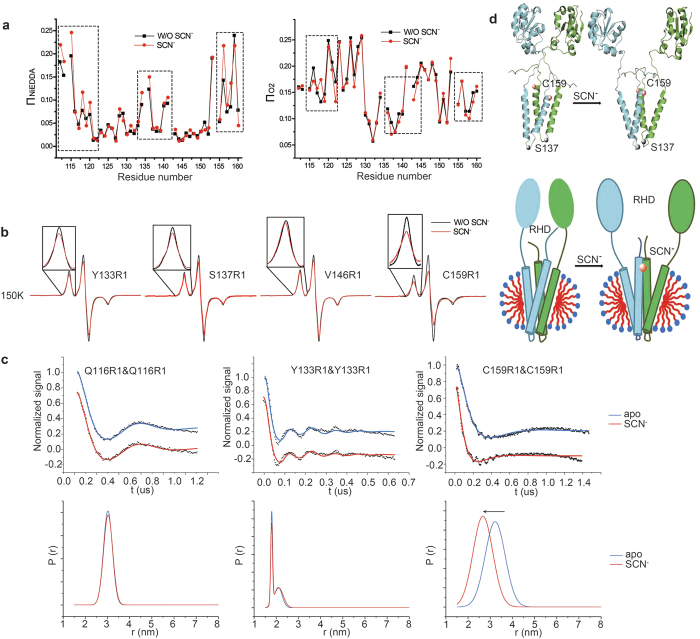
Conformation rearrangement analysis of full-length YgaP upon SCN^−^ binding. (**a**) Deviation analysis of accessibility parameters Π_NiEDDA_ (left) and Π_O2_ (right) between the YgaP samples in the absence or presence of SCN^−^ plotted versus the residue number. (**b**) CW-EPR spectral overlap of the YgaP variants (Y133R1, S137R1, V146R1, and C159R1) (black line) in the presence of SCN^−^ (red line) at 150 K. (**c**) Distance measurements of YgaP variants(Q116R1, Y133R1 and C159R1) using DEER method in the absence (blue) or presence of SCN^−^ (red). In the top panels, background corrected experimental data and best fit by DEFit-v4.1(colored solid line) are presented, the corresponding obtained distance distributions are shown in the lower panel. (**d**) Hypothetical conformational changes of YgaP upon SCN^−^ binding (top) and corresponding topological model in DPC micelles (bottom). The rhodanese domain is represented by an ellipsoid, and the transmembrane helices are represented by cylinders. Each monomer of YgaP is colored cyan and green.

Based on the lower mobility of YgaP-TMH2 observed in the presence of SCN^−^, conformational rearrangements of YgaP-TMD were also analyzed using accessibility analysis. The accessibility parameters, Π_O2_ and Π_NiEDDA_, were obtained using EPR power saturation experiments and are shown in Fig. 5a. Similar to the mobility data, accessibility for the majority of the residue sites was not affected by the addition of SCN^−^. However, dramatic changes in accessibility were observed in the residue sites located in three different regions of the YgaP-TMD: the N-terminus of TMH1 (P112R1, R115R1, I119R1, A120R1, and L113R1), the loop region (T134R1, N136R1, and L141R1) and the second half of TMH2 (from S156R1 to G160R1) (dashed box in Fig. 5a). In these regions, the Π_NiEDDA_ values increased, whereas the Π_O2_ values decreased in the presence of SCN^−^, strongly indicating increased exposure of this area to aqueous buffer upon SCN^−^ binding.

Because distance change between residues is the most direct evidence of protein conformational rearrangement, low-temperature CW-EPR experiments for several residues were also conducted to detect the spectral broadening caused by dipolar coupling from two approaching spins. As shown in Fig. 5b, the CW-EPR spectra of Y133R1, S137R1, V146R1 and C159R1 showed broadening upon SCN^−^ binding at low temperatures (150 K), where the motion effect is eliminated, indicating that the broadening resulted from approaching the inter-residue distance between two identical residues from each monomer. However, no apparent spectral broadenings were observed for the other residue sites in the transmembrane domain in the presence of SCN^−^ (data not shown). As highlighted in the structure of full-length dimeric YgaP in Fig. 5b, the residues with distance variation upon SCN^−^ binding (Y133R1, S137R1, V146R1 and C159R1) reside in two different regions; residues Y133R1 and S137R1 are located on the loop between the two transmembrane helices, whereas the other residues are located in the second transmembrane helix. The calculated full-length dimeric YgaP structure (Fig. 3c) demonstrates that the spectral broadening observed at residue sites V146 and C159 was caused by decreased distance, indicating tight packing of YgaP-TMH2 induced by SCN^−^ binding. Furthermore, DEER experiments for YgaP variants Q116R1, Y133R1 and C159R1, which reside in different regions in YgaP-TMDs, were performed to analyze conformational changes of YgaP upon SCN^−^ binding. As shown in Fig. 5c, distance distributions between the two spins of YgaP variants Q116R1, Y133R1 and C159R1 in the absence or presence of SCN^−^ were derived from DEER data. Overall, significant changes of distance distribution induced by SCN^−^ binding exhibited only in YgaP C159R1, with average distance between two spins decreased from 3.22 nm to 2.67 nm. And the fraction of short distance (<2.50 nm) in distance distribution increased significantly with the presence of SCN^−^, which was consistent with CW-EPR data in Fig. 5b. Although SCN^−^ binding didn’t change the distance distribution at residue site Y133, two distance components between spin pairs were observed, demonstrating that two major different conformational state may exist of the Y133 side chain. It’s quite reasonable because Y133 reside in the end of TMH1, near the high flexible loop region. Additionally, the distance distribution of YgaP C159R1 decreased from 1.45 nm to 1.33 nm upon binding of SCN^−^, which indicated the mobility decrease as described before.

Taken together, the accessibility data and the EPR-derived distance information demonstrated an SCN^−^ induced conformational change in the second half of YgaP-TMH2. Because of the decreased distance between the two C159 residues and the increased solvent accessibility in this area, the second half of YgaP-TMH2 from each monomer subunit may pack together upon SCN^−^ binding, as illustrated in Fig. 5d. The approach between the two YgaP-TMH2s from each monomer brought the two C-termini of TMH2 into close proximity, thus resulting in the broadening of the spectrum of spin label at residue C159 upon SCN^−^ binding. In the previous solution NMR titration studies of the YgaP-TMD, C159 was reported to be near the binding site of SCN^−^ 21. Furthermore, the accessibility changes of most residues in the N-terminus of YgaP-TMH1 and the second half of YgaP-TMH2 upon SCN^−^ binding implied increased aqueous accessibility.

## Discussion

YgaP is the only member of the rhodanese family in *E. Coli*, locating in the plasma membrane and YgaP is hypothesized to export non-toxic thiocyanate (SCN^−^) across the membrane. The tertiary structure of dimeric full-length YgaP was still unknown in the NMR studies reported recently^21^, owing to the lack of long distance constrains between the transmembrane domain and cytosolic catalytic rhodanese domain. EPR distance measurements (8–25 Å for CW-EPR and 20–80 Å for DEER-EPR) can provide broader distance information than NMR, leading to a better converged global structure fold for large- or multiple-domain proteins^38^,^39^. To characterize the structure of *E. coli* integral membrane protein YgaP in detergent micelles, systematic site-specific EPR mobility and accessibility analyses were conducted to derive secondary structures of the YgaP-TMD. The dimeric YgaP-TMDs structure determined using EPR derived distance constraints illustrated that the two transmembrane helices formed the interface of the homodimer. Additionally, long-distance constraints measured by DEER demonstrated that the YgaP rhodanese domain was connected to the TMDs by a long flexible linker, forming a dimeric full-length YgaP structure. This combined approaches of continue-wave, pulsed EPR and rigid-body computation we presented here can provide a novel pathway to characterize the structure of other membrane proteins.

Moreover, based on the dimeric full-length YgaP structure, combined EPR methods were applied to provide insights into the SCN^−^ binding mechanism. The mobility decrease induced by SCN^−^ binding was discovered at residue sites predominantly in the second transmembrane helix, suggesting that tighter packing occurred at YgaP-TMH2 relative to the apo- state. Besides, regions involved in the SCN^−^ induced conformational change were mapped to the C-terminus of TMH2 through EPR-derived distance and accessibility analyses. Upon SCN^−^ binding, residues in this region (particularly those in the vicinity of Cys 159) exhibited decreasing distance, indicating a tighter packing between the TMH2 regions from each monomer, which was consistent with the structure change discovered by Eichmann *et al.*^21^. Additionally, the aqueous solvent exposure increase caused by SCN^−^ binding was also observed in the first half of YgaP-TMH1(from P112 to G123), which is likely because the TMH1 regions are moving away from one another (Fig. 5c). Taken together, the results suggest that YgaP is involved in SCN^−^ binding and exportation. A tighter packing state of YgaP-TMH2 may be a consequence of the conformational rearrangement and mobility changes induced by SCN^−^ binding, which demonstrated that the second transmembrane helix of YgaP played a crucial role in SCN^−^ exportation. Further functional analysis and spectroscopic experiments are required to elucidate the entire exportation mechanism.

## Methods

### Construction of site-directed cysteine mutations of full-length YgaP

The cysteines at sites 64 and 159 in wild-type full-length YgaP from *E. coli* K-12 were mutated to serines using the PCR-driven overlap extension method, resulting in a Cys-less construct. Then, 45 single- (P112-G160, except M114, V135, L142, and G154) and 17 double-cysteine mutants of full-length YgaP were constructed through site-directed mutagenesis. Cys-less and mutant YgaP sequences were inserted into expression vector p28 (modified from pET-28a, Novagen) using the *Nde I* and *Not I* restriction sites. All cloning constructs were verified through sequencing.

### Protein expression and purification

The recombinant plasmids were transformed into the *E. coli* BL21-Gold (DE3) expression strain and grown in M9 media at 25 °C. At a cell density of OD_600_ = 0.8, protein over-expression was induced with a final concentration of 0.8 mM isopropyl-D-thiogalactoside (IPTG), followed by 20 h of shaking. The YgaP proteins were expressed in the cellular membrane fraction. The bacterial cells were harvested using high-speed centrifugation (Allegrax-15R, Beckman) at 4,000 g for 20 min. Then, the cell pellets were suspended using lysis buffer (70 mM Tris, 300 mM NaCl, pH 8.0) for cell lysis using probe sonication. Next, the expressed YgaP proteins were extracted from the membrane fraction using buffers containing 1% (w/v) LDAO detergent. The solubilized proteins were purified using Ni-NTA affinity chromatography (QIAgen, Germany) and eluted using buffer (20 mM Tris, 200 mM NaCl, 2 mM dithiothreitol (DTT), pH 8.0) containing 0.2% DPC. Sodium dodecyl sulfate polyacrylamide gel electrophoresis (SDS-PAGE) was applied to analyze the purified YgaP proteins in DPC micelles.

### Circular dichroism (CD) spectroscopy analysis

YgaP proteins in DPC micelles were diluted to 0.10 mM in 50 mM NaH_2_PO_4_-Na_2_HPO_4_ buffer with DPC detergent, pH 8.0. CD spectra were collected on a Jasco-810 spectropolarimeter at 298 K. All spectra were recorded over a wavelength range of 190–280 nm using a cuvette of 1 mm path length at a scanning speed of 20 nm/min and subjected to 10 scans. Acquired data were normalized by subtracting the baseline recorded for the buffer only and are shown in the supplementary data (Supplementary Figure 2).

### 1-Oxyl-2,2,5,5-tetramethyl-Δ3-pyrr-oline-3-methyl methanethiosulfonate (MTSL) spin labeling

The MTSL spin radical (Toronto Research Chemicals, Ontario, Canada) was introduced into the thiol groups of each cysteine mutant with a spin label/protein molar ratio of 10:1 at 4 °C, overnight. Excess spin reagents were removed using a PD-10 gravity flow desalting column (GE Biosciences). The labeled protein was finally eluted in binding buffer (20 mM Tris, 200 mM NaCl, 2 mM DTT, pH 8.0) with 0.2% (w/v) DPC. The labeling efficiency for each mutant was analyzed by measuring the protein concentration and the spin concentration, and the efficiency was approximately 90%. Single cysteine YgaP variants were concentrated to approximately 200 μM using Amicon Ultra-15 centrifugal filter units (Millipore) for further EPR experiments. The MTSL-labeled YgaP dimer was dissociated to monomers using a buffer (20 mM Tris, 200 mM NaCl, pH 8.0) containing 0.2% (w/v) SDS. Then, the monomeric YgaP solution was mixed with 10-fold molar excess of unlabeled Cys-less protein. The mixed solution was incubated for 1 hour before it was loaded onto Ni-NTA resin. After detergent exchange from 0.2% SDS to 0.2% DPC, the refolded YgaP dimers were verified by SDS-PAGE (Supplementary Figure 1b). The final spin concentration was maintained at approximately 200 μM.

### Continuous wave EPR (CW-EPR) spectroscopy

CW-EPR spectra were acquired using a Bruker A300 spectrometer (Bruker Biospin GmbH, Rheinstetten, Germany) at X-band (9.5 GHz) equipped with a high-sensitivity cavity (ER 4119HS, Bruker Biospin GmbH, Rheinstetten, Germany). The samples were placed into a quartz capillary tube (approximately 0.5 mm, Kimble micro-capillary pipets) with a volume of approximately 20 μL. Mobility and power saturation analyses were conducted using the CW-EPR spectra of YgaP with single MTSL labeling acquired at 298 K. CW-EPR spectra were acquired with a 100 kHz modulation frequency, 2 mW incident microwave power (20 dB attenuation), 1 Gauss modulation amplitude, 10.24 ms time constant, 40.96 ms conversion time and 150 Gauss scan width. The inverse line width of the central m_I=0_ resonance line (ΔH^−1^) of the CW-EPR spectrum was extracted by the Peak_fit.exe software from the Labview EPR program developed in Dr. Piotr Fajer’s laboratory.

For distance measurements using CW-EPR, the spectra of YgaP with single or double MTSL labeling were all acquired at low temperature (150 K). Under this low temperature condition, 40% glycerol was added as a cryoprotective agent. The CW-EPR spectra were acquired at 150 K with similar parameters, except a 7.73 × 10^−2^ mW incident microwave power (35 dB attenuation) and a 200 Gauss scan width. The spin-spin distances of the double labeled protein were analyzed using the Monte Carlo/Simplex Gaussian convolution method using the CWdipFit software to deconvolute the spectral broadening of the double labeled samples compared to the sum spectra of single labeled samples.

For the power saturation studies and accessibility analysis, the experiments were performed using the same spectrometer equipped with an ER4123D CW resonator (Bruker BioSpin, Germany). Samples with a total volume of ~3 μL were loaded into gas permeable TPX capillary tubes. During the power saturation analysis, the incident microwave power ranged from 0.7 mW (25 dB attenuation) to 180 mW (1 dB attenuation) in 2-dB steps. To obtain the EPR spectra of the YgaP sample in an N_2_ atmosphere, the O_2_ atmosphere of the sample was purged by N_2_ blowing or equilibration. Simultaneously, EPR spectra were acquired in air (21% O_2_ as the hydrophobic paramagnetic reagent) or in an N_2_ atmosphere in the presence of the 50 mM Ni^2+^-EDDA (NiEDDA) complex as the hydrophilic paramagnetic reagent. Power saturation curves were measured as the vertical peak-to-peak amplitude (A) of the first derivative M_i=0_ line as a function of incident microwave power (P). To determine the value of P_1/2_, the data were fitted using an R software script based on the equation: 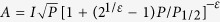, where I is a scaling factor and P_1/2_ is the incident power when the first derivative amplitude is half of what it would be if unsaturation occurred. With the obtained P_1/2_, accessibility parameters Π_O2_ and Π_NiEDDA_ were calculated according to the equation:


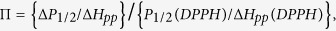


where ΔP_1/2_ was calculated between two P_1/2_ values (presence and absence of relaxing agent), ΔH_pp_ is the peak-to-peak line-width of the first derivative spectrum, and P_1/2_(DPPH) and ΔH_pp_(DPPH) were the P_1/2_ and line-width values, respectively, of a standard sample of crystalline 2,2-diphenyl-1-picrylhydrazyl (DPPH) in KCl.

### Pulsed EPR experiments

Double electron-electron resonance (DEER) experiments were performed with a Bruker Elexsys E680 spectrometer (Bruker Biospin, Billerica, MA) at X-band (9.7 GHz) using a four-pulse sequence at 65 K. The observed pulse lengths of π/2 and π were 24 and 48 ns, respectively, and the pump pulse length was 24 ns. A 65 MHz frequency offset was used for the pump pulse. Glycerol (30–40%) was added to the samples as a cryoprotectant. All DEER spectra were analyzed with a Monte Carlo/Simplex Gaussian convolution method using the DEFit-v4.1 software.

### Distance constraints preparation

All distances between spin-labeled side chains were measured by CW-EPR and DEER. These data were converted into a distance range between two corresponding C_β_ atoms by a simple “motion on a cone” model. Before computational modeling of the YgaP proteins, the energy function term for the EPR distance constraints was constructed by referring to the solution NMR NOE distance constraints. The distance constraints were also presented with different orientations of the two spin labels, the minimum distance of corresponding C_β_ atoms (d_Cβ_) was d_SL _− σ_SL_ − 12.5 Å and the maximum distance was d_SL_ + σ_SL_ + 2.5 Å, where d_SL_ is the distance between two spin labels and σ_SL_ is the standard deviation of d_SL_. All distance constraints were presented in Supplementary Table 1. The minimum and maximum distances of d_Cβ_ were further used for structure refinement.

### Computational modeling using XPLOR-NIH

For the preparation of the monomeric YgaP transmembrane domain model, two transmembrane bundles of monomeric YgaP were initially prepared following the helical secondary structure determination from the periodic oscillation of the EPR central peak mobility or accessibility analyses. Then, intra-monomeric EPR distance constraints were applied for simulated annealing calculations, which resulted in a rigid-body model of the two-helix transmembrane domain of monomeric YgaP. During these calculations, the conjoined rigid body/torsion angle/Cartesian dynamics simulated annealing protocol was used, in which the rigid bodies were allowed to translate and rotate freely while staying connected by a flexible linker with Cartesian degrees of freedom. First, a 1,000-step initial minimization was performed, followed by a long high temperature simulation for 800 ps or 8,000 variable time steps (whichever came first) at 3000 K with constraints from the experimental EPR distance constraints, bonds, angles, dihedral angles (within the linker), improper dihedral angles, van der Waals (VDW) repulsion, and torsion angle database potentials. During the high-temperature simulations, only Cα atoms were used to compute the non-bonded energies, with a radius scale factor of 1.2 and a force constant of 0.004 kcal mol^−1^.Å^−4^. After the high-temperature simulation, a simulated annealing step was performed, during which the temperature was finally decreased to 25 K in temperature steps of 25 K. At each temperature, the system was balanced for 1 ps or 200 steps (whichever came first). During simulated annealing, the following parameters were ramped: VDW radius scale factor, from 0.9 to 0.8; E_repel_ force constant, from 0.004 to 4 kcal.mol^−1^.Å^−4^; E_RAMA_ force constant, from 0.002 to 1; bond angle force constant, from 200 to 500 kcal.mol^−1^.rad^−2^; improper angle force constant, from 50 to 500 kcal.mol^−1^.rad^−2^; and torsion angle constraint force constant, from 0.1 to 200 kcal.mol^−1^.rad^−2^. The following parameters were fixed: 1,000 kcal.mol^−1^ distance force constant and 1,000 kcal.mol^−1^.Å^−2^ bond force constant. The final structure model was obtained after 200 steps or 1 ps Powell gradient minimization.

For the computational modeling of the dimeric YgaP transmembrane domains, the structural model was constructed by translating and rotating the two monomeric models to ensure that the two chains were identical. Inter-monomeric EPR distance constraints were applied for simulated annealing calculations, resulting in a converged structural model of the dimeric YgaP transmembrane domains. During the calculation procedure for the dimeric YgaP transmembrane domains, each monomer was considered a rigid body, and they were allowed to translate and rotate with respect to one another. The refinement procedure was similar to that of the single-chain model, except the following: (1) in the high-temperature simulation step, the high temperature was 360 K; and (2) in the simulated annealing step, the temperature steps were set at 6 K, and the final temperature was also 6 K.

For computational modeling of the dimeric full-length YgaP protein, the calculation procedure was similar to that of the dimeric YgaP transmembrane domains. An initial model was constructed using a linker region between the cytosolic domain (which was determined using solution NMR) and each monomer of the YgaP transmembrane domain using MODELLER^40^. Then, the EPR distance constraints between two identical residues in the two cytosolic domains and the distance constraints between two residues in the cytosolic and transmembrane domains were applied for the full-length YgaP modeling. The parameters of the computational procedure were the same as those used in the simulated annealing protocol for the monomeric YgaP-TMD.

### Enzymatic product (SCN^−^) binding to dimeric full-length YgaP

NaSCN (Sigma-Aldrich; final concentration of 20 mM) was added to the spin-labeled dimeric full-length YgaP in DPC micelles and then CW-EPR spectra were acquired at 298 K. Subsequent CW-EPR mobility and power saturation analyses were performed at each residue site in the presence or absence of SCN^−^. The CW-EPR spectra of several spin labeled variants (I119R1, Y133R1, S137R1, V146R1, A148R1, G149R1, and C159R1) were also acquired at 150 K, either in the presence or absence of SCN^−^.

## Additional Information

**How to cite this article**: Ling, S. *et al.* Structure of an *E. coli* integral membrane sulfurtransferase and its structural transition upon SCN^−^ binding defined by EPR-based hybrid method. *Sci. Rep.* **6**, 20025; doi: 10.1038/srep20025 (2016).

## Supplementary Material

Supplementary Information

## Figures and Tables

**Table 1:** Statistics of structure computations for 16 total dimeric full-length YgaP.

	Full-length (dimer)
**EPR distance constraints**	
Distance constraints	
Total	47
Intra-molecule	34
Inter-molecule	13
Deviations from idealized geometry	
Bond lengths (Å)	0.022
Bond angles (^o^)	2.488
Impropers (^o^)	4.993
Average pairwise r.m.s. deviation (Å)^a^ of Cα	1.59
**Structure statistics**	
Violations (mean and s.d.)	
Distance constraints (Å)	0
Max. distance constraint violation (Å)	0

^a^Pairwise r.m.s. deviation was calculated among top 16 refined structures.
