# Supplementary material for: Structure of an E. coli integral membrane sulfurtransferase and its structural transition upon SCN− binding defined by EPR-based hybrid method
Source: Sci Rep. 2016 Jan 28;6:20025. doi: 10.1038/srep20025 (PMC4730233; doi:10.1038/srep20025)
Supplement: Supplementary Information [file srep20025-s1.pdf]

Structure of an *E. coli* integral membrane sulfurtransferase and its structural transition upon SCN^- binding defined by EPR-based hybrid method

Shenglong Ling[1,],Wei Wang[1], Lu Yu[1,], Junhui Peng[1], Xiaoying Cai[1], Ying Xiong[1], Zahra Hayati[2], Longhua Zhang[1], Zhiyong Zhang[1,]*, Likai Song[2,]*, Changlin Tian[1,]*

[1.] National Laboratory for Physical Science at Microscale, School of Life Science, University of Science and Technology of China, and High Magnetic Field Laboratory, Chinese Academy of Sciences, Hefei, 230027, P. R. China.

[2.] National High MagneticField Laboratory, Florida State University, Tallahassee, FL, 32310, U.S.A.

*Correspondence should be addressed to Z.Z. (zzyzhang@ustc.edu.cn),L.S. (song@magnet.fsu.edu) or C.T.(cltian@ustc.edu.cn)

Supporting Figures and Tables:

Figure S1.(a)Primary sequence and topology diagram of YgaP. The topology diagram of YgaP rhodanese domain(blue) was derived from solution NMR structure (PDB:2MRM), while two transmembrane helices (orange cylinder)were predicted by software TMHMM. Residues selected for site-directed spin labeling (P112 to G160) were highlighted by red box. (b) SDS-PAGE analysis of YgaP in DPC micelles. Lane 1 was protein markers, YgaP L113C in DPC micelles sit on Lane 2 showed a monomer state, while cross-linked YgaP L113C in Lane 3 indicated a dimeric state with a molecular weight about 40 kD in DPC micelles. Line 4 showed that the dissociated YgaP L113C monomers can refold into dimers after detergent exchange from 0.2% SDS to 0.2% DPC.

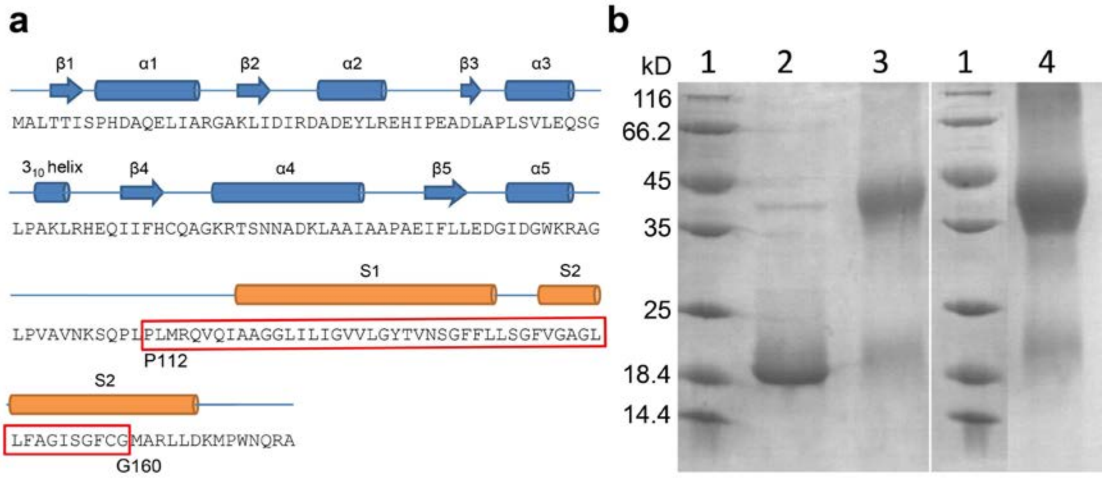

Figure S2. Circular dichroism spectra of wild-type YgaP, cysless variant YgaP C64S & C159S and double cysteines variant YgaP A120C & S143C in DPC micelles.

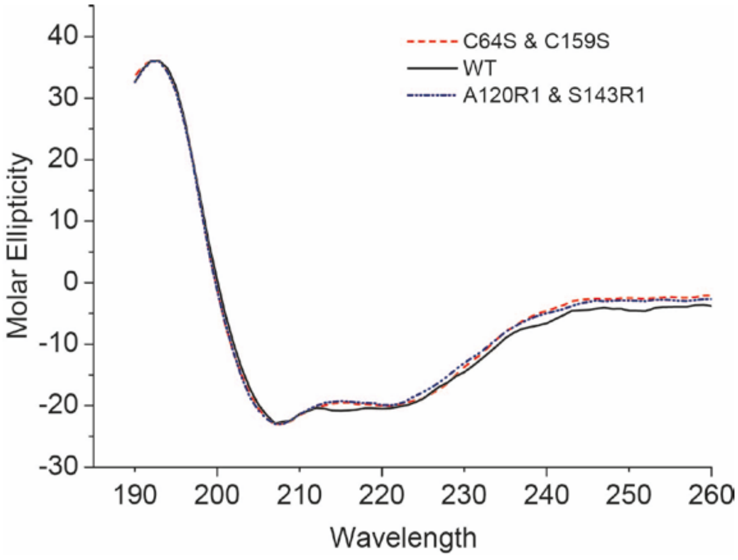

Figure S3. EPR power saturation curves for YgaP L124R1 in DPC micelles. The power saturation curves were collected under three conditions: molecular oxygen (O_2), nitrogen (as a control) and the reagent NiEDDA (50 mM). Π_{O2} and Π_{NiEDDA} values from fitting were presented.

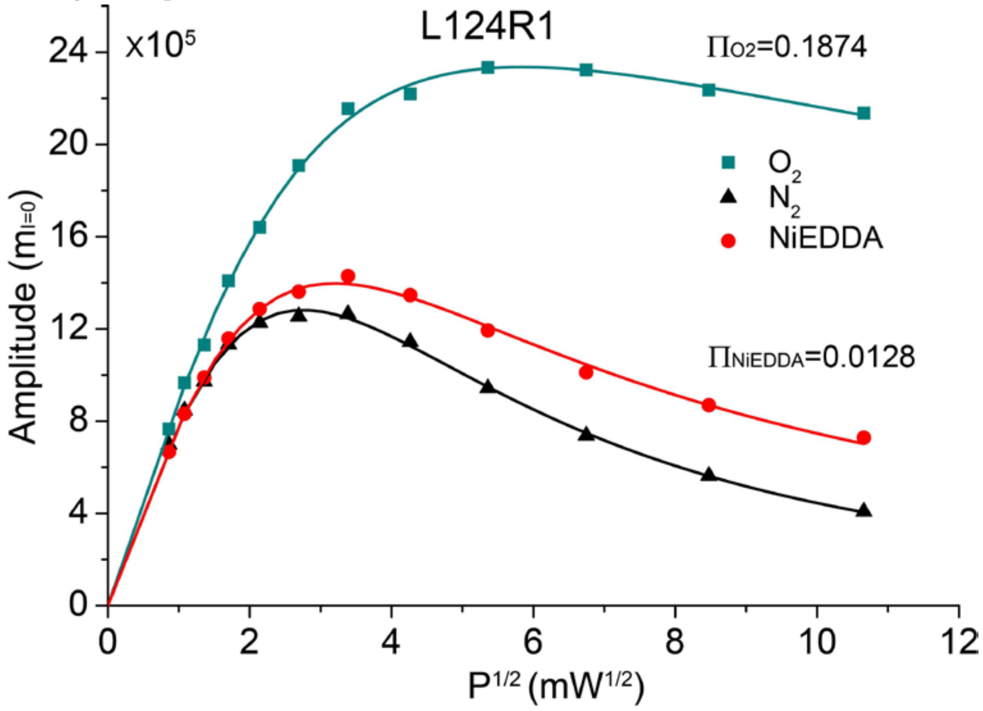

Figure S4. Membrane immersion depth parameters (ϕ) of sequential residues in TM domain derived from accessibility parameter were plotted versus the residue number.

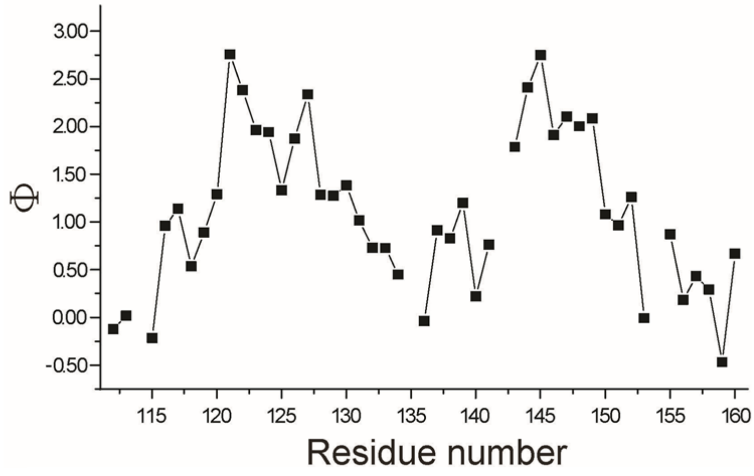

Figure S5. Intra-monomeric CW-EPR distance measurements of double labeled YgaP samples:I127R1/F139R1, I127R1/S143R1, I127R1/A148R1 and V135R1/A148R1 (both residues located on the same monomer of YgaP variants) at 150K. The black line was sum of spectra with two single-labeled spins and the red line was the spectrum of double-labeled spins.The average inter-spin distance between two residues was shown besides the spectrum.

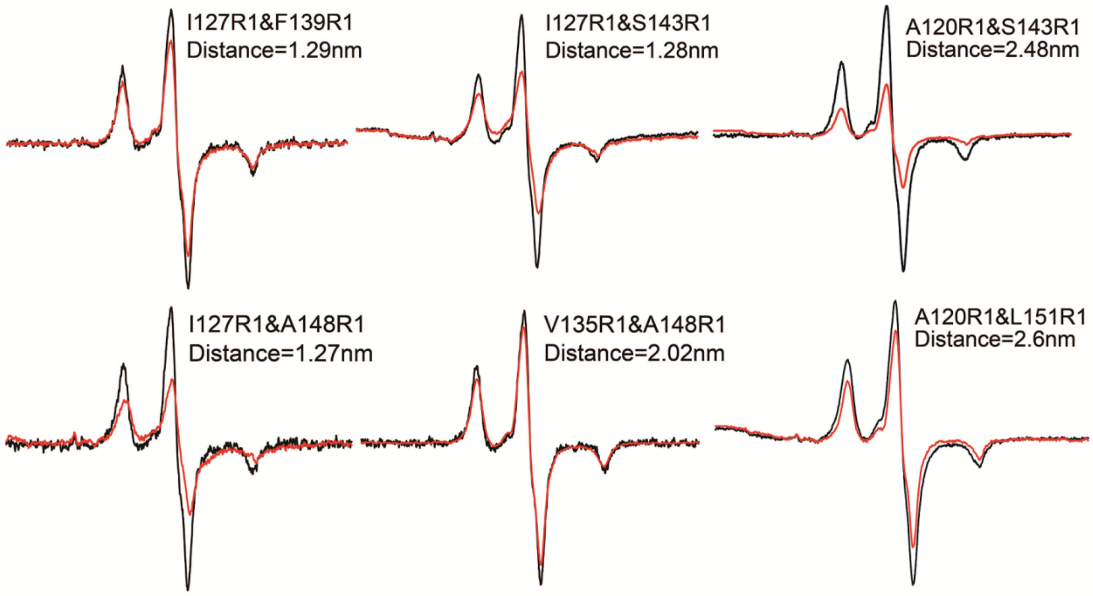

Figure S6. Comparison of monomeric YgaP-TMD structure determined in this report (cyan) and the solution NMR structure of YgaP-TMD by Eichmann et al.(Green, PDB code 2MPN)

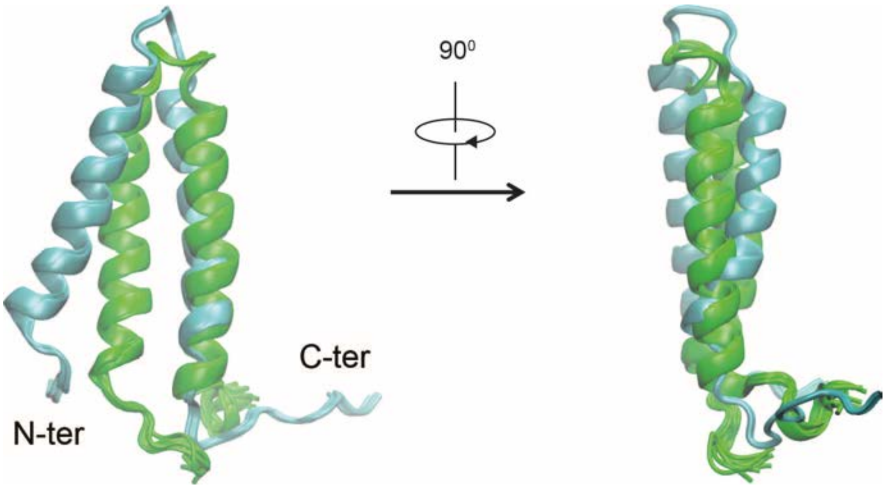

Figure S7. Inter-monomeric DEER-EPR distance measurements between two same spin labeled residues (S49R1, I80R1, S108R1, L124R1, V130R1, F139R1, F145R1,G147R1 and I155R1) on each monomer of dimeric YgaP variants, at 65 K. The red line was the best fit of the normalized signal versus dipolar evolution time. The insets in the upper right of each DEER spectrum represented the average distance.

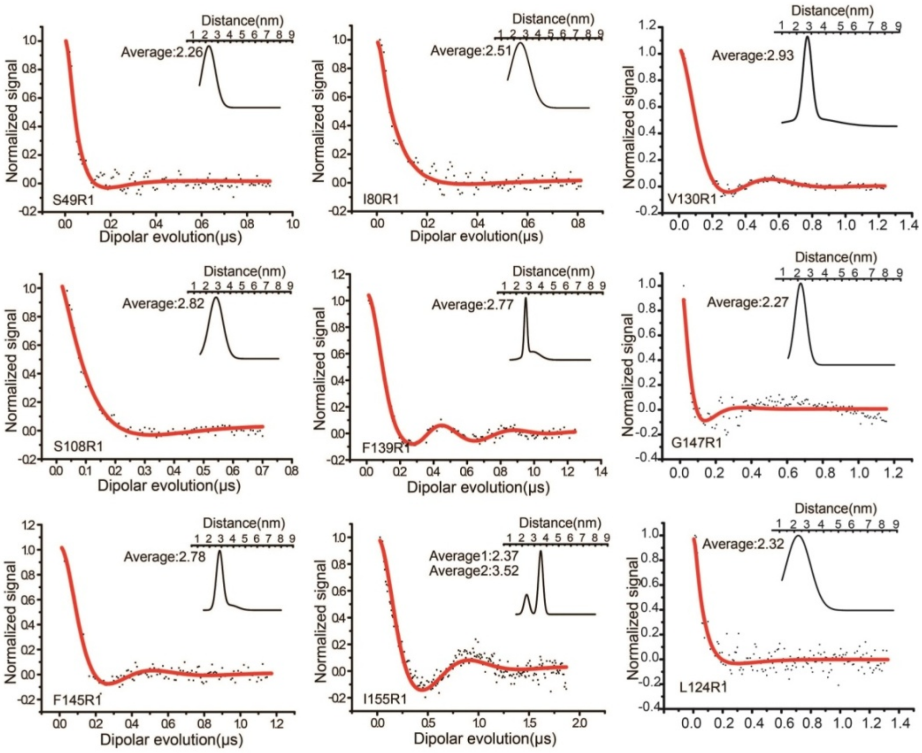

Figure S8. The flow chart showing structural model refinement of dimeric full-length YgaP using software Xplor-NIH.

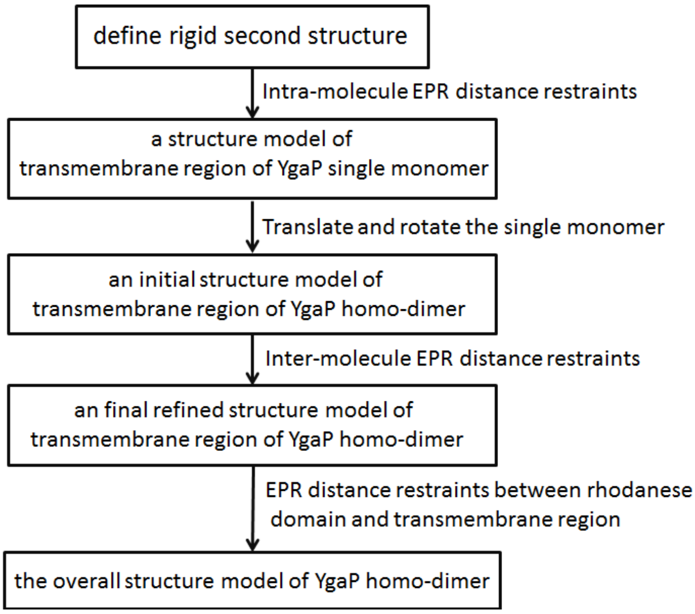

Figure S9. DEER-EPR distance measurements of double spin labeled samples: S49R1/Q116R1, S49R1/N136R1 and S49R1/L151R1 at 65 K. Two spin labels were located on rhodanese domain and TMD of a single YgaP monomer. The red line was the best fit of the normalized signal versus dipolar evolution time. The insets in the upper right of each DEER spectrum represented the average distance.

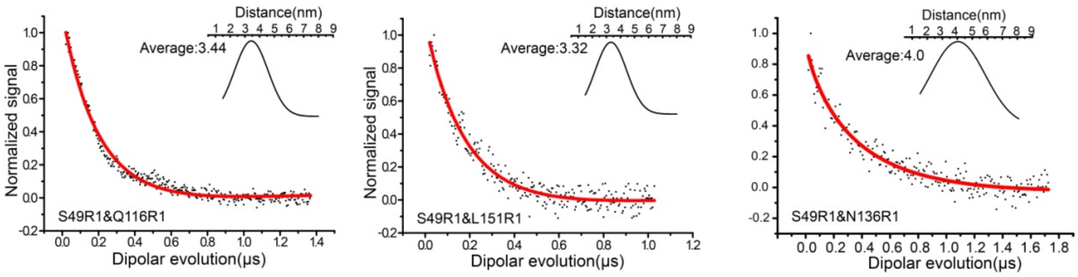

Figure S10. Superposition of the final 16 conformers of dimeric full-length YgaP determined in this report.

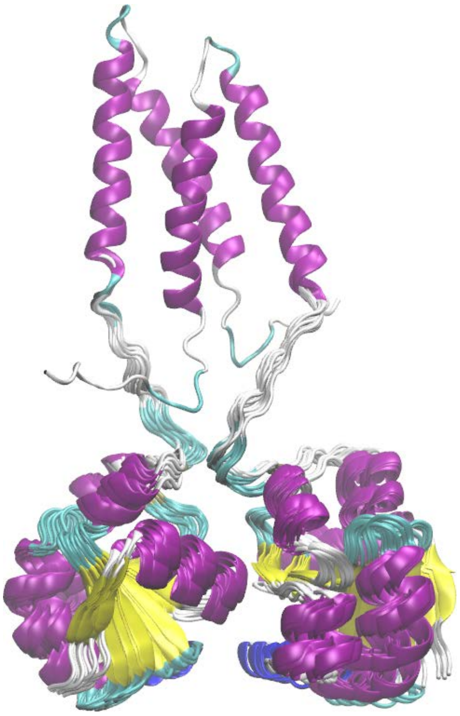

Figure S11. CW-EPR spectra scanning of 45 spin labeled YgaP mutants (range from Pro112 to Gly160, except M114, V135, L142, G154) in the transmembrane domain in the presence of SCN^- at 298 K. Each spectrum was normalized by the height of the central peak.

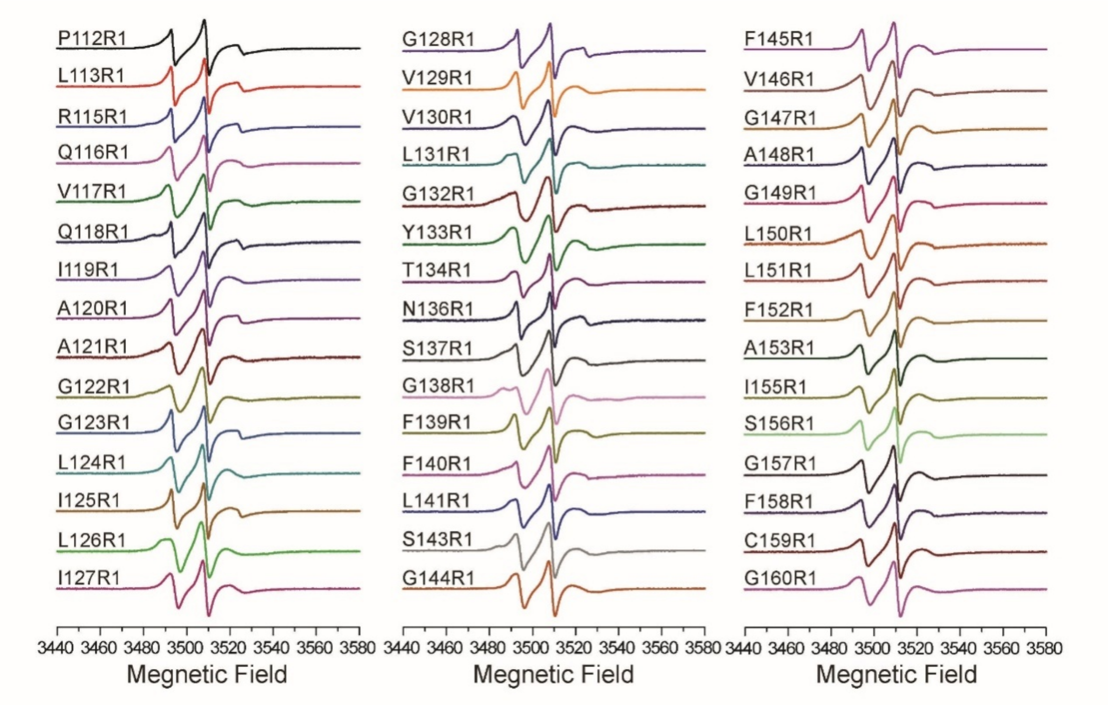

Table S1.EPR distance constrains for YgaP structural model refinement

**EPR distance constraints**	**D_{SL} [a]**	**σ_{SL} [b]**	**D_{SL}+2.5+σ_{SL} [c]**	**D_{SL}-σ_{SL}-12.5 [d]**
Intra –molecular (Å)				
CW-EPR distance constraints				
A120-I127	15.9	7.35	25.75	0
A120-S143	24.8	4.22	31.52	8.08
A120-L151	26	7.29	35.79	6.21
I127-F139	12.9	7.64	23.04	0
I127-S143	12.8	7.1	22.4	0
I127-A148	12.7	8.3	23.5	0
V135-A148	20.2	12.7	35.4	0
A148-I155	12.8	14	29.3	0
DEER distance constraints				
A120-V135	25.7	10.1	38.3	3.1
A120-F139	25.2	5.45	33.15	7.25
A120-I155	30.5	5.83	38.83	12.17
F139-I155	31.1	8.78	42.38	9.82
S49-Q116	34.4	26.9	86.3	0
S49-N136	40.0	42.3	84.8	0
S49-L151	33.2	26.4	62.1	0
D94-Q116	35.7	30.0	68.2	0
D94-L151	32.8	28.0	63.3	0
Inter –molecular (Å)				
DEER distance constraints				
S49-S49	22.6	12.5	37.6	0
C64-C64	27.1	12.3	41.9	2.3
I80-I80	25.1	18.4	46.0	0
D94-D94	25.6	11.1	39.2	2.0
S108-S108	28.2	13.6	44.3	2.1
Q116-Q116	30.2	8.5	41.2	9.2
L124-L124	23.2	17.9	43.6	0
V130-V130	29.3	5.87	37.67	10.93
T134-T134	30.7	3.3	36.5	14.9
F139-F139	27.7	2.75	32.95	12.45
F145-F145	27.8	0.792	31.092	14.508
G147-G147	22.7	8.13	33.33	2.07
I155-I155	23.7	4.93	31.13	6.27

[a] Distance between two spin radicals labels.

[b] Standard deviation of D_{SL} .

[c] Maximum Cβ distance predicted according D_{SL}.

[d] Minimum Cβ distance predicted according D_{SL}.

Table S2. Structural modeling statistics for top 20 protein structures from total 96 computed structures of monomeric YgaP-TMDs.

	**TMD (monomer)**
**EPR distance constraints**	
Distance constraints	
Total	12
Intra-molecule	12
Inter-molecule	0
Deviations from idealized geometry	
Bond lengths (Å)	0.001
Bond angles (^{o})	0.373
Impropers (^{o})	0.232
Average pairwise r.m.s. deviation (Å) [a] of Cα	19.8
**Structure statistics**	
Violations (mean and s.d.)	
Distance constraints (Å)	0
Max. distance constraint violation (Å)	0

[a] Pairwise r.m.s. deviation was calculated among top 20 refined structures.

Table S3. Structural modeling statistics for top 100 protein structures from total 1,000 computed structures of dimeric YgaP-TMDs.

	**TMD (dimer)**
**EPR distance constraints**	
Distance constraints	
Total	33
Intra-molecule	24
Inter-molecule	9
Deviations from idealized geometry	
Bond lengths (Å)	0.004
Bond angles (^{o})	0.464
Impropers (^{o})	0.393
Average pairwise r.m.s. deviation (Å) [a] of Cα	1.81
**Structure statistics**	
Violations (mean and s.d.)	
Distance constraints (Å)	0
Max. distance constraint violation (Å)	0

[a] Pairwise r.m.s. deviation was calculated among top 100 refined structures.
